# Prevalence of blindness and causes of visual impairment among adults aged 50 years or above in southern. Jiangsu Province of China

**DOI:** 10.12669/pjms.295.3866

**Published:** 2013

**Authors:** Yong Yao, Jun Shao, Wei Sun, Jing Zhu, Dong Hong Fu, Huaijing Guan, Qinghuai Liu

**Affiliations:** 1 Yong Yao, Orthopedics Department, The Affiliated Hospital of Shandong Traditional Chinese Medicine University,Jinan, China.; 2 Jun Shao, Department of Ophthalmology, Nanjing Medical University Affiliated the Wuxi People’s Hospital, Qing Yang Road 299, Wuxi 214023, China.; 3 Wei Sun, Department of Ophthalmology, Suzhou Municipal Hospital, Daoqian Road 26, Suzhou 215008, China.; 4 Jing Zhu, Department of Ophthalmology, Nanjing Medical University Affiliated the Wuxi People’s Hospital, Qing Yang Road 299, Wuxi 214023, China.; 5 Dong Hong Fu, Department of Ophthalmology, Nanjing Medical University Affiliated the Wuxi People’s Hospital, Qing Yang Road 299, Wuxi 214023, China.; 6 Huaijing Guan, Department of Ophthalmology, Affiliated Hospital of Nantong University, Nantong 226001, China.; 7 Qinghuai Liu, Orthopedics Department, The Affiliated Hospital of Shandong Traditional Chinese Medicine University,Jinan, China.

**Keywords:** Blindness, Epidemiology, Eye diseases, Low Vision

## Abstract

***Objective: ***The prevalence of blindness and low vision among adults aged ≥50 years in southern Jiangsu Province were surveyed and estimated.

***Methods: ***Cluster sampling was employed from January to September 2010 to randomly select 6,722 individuals aged ≥50 years in 28 clusters from southern Jiangsu Province. The survey was preceded by a pilot study, which refined operational methods and conducted quality assurance evaluation. Eligible individuals were registered for visual acuity measurement and eye examination.

***Results: ***A total of 6,155 individuals were recruited, and a response rate of 91.50% was obtained. The prevalence of bilateral blindness and low vision were found to be 0.76% and 1.37%, respectively. Subjects with monocular blindness and low vision were 3.27% and 3.48%, respectively. Among the individuals evaluated, 201 were detected to have monocular blindness and 47 with bilateral blindness. In addition, 55 of the 201 subjects with monocular blindness were found to suffer from low vision of the other eye. Among the 295 subjects with blind eyes, 116 (39.32%), 31 (10.51%), and 28 (9.49%) were caused by cataract, high myopia macular degeneration, and atrophic eyeballs, respectively. In the 437 subjects with low-vision eyes, 223 (51.03%), 41 (9.38%), and 41 (9.38%) had cataract, high myopia macular degeneration, and age-related macular degeneration, respectively.

***Conclusions:*** Blindness and low vision are caused by descending cataract, age-related macular degeneration, high myopia macular degeneration, and atrophic eyeballs.

## INTRODUCTION

Low vision and blindness are two of the most significant health and socioeconomic risks in developing countries. China, known for having the world’s largest population, contributes substantially to global blindness.^1^ With population growth and increasing life expectancy, the magnitude of blindness is expected to increase further.^1^ Blindness and low vision are recently reported to have affected approximately 5.8% Chinese adults aged older than 50 years.^2^

The large-scale eye disease epidemiological survey in China could be traced to the 1980s. Several epidemiological investigations on low vision and blindness were conducted in different provinces. The prevalence of visual impairment (VI), especially blindness, varies greatly based on the developmental status of the geographic region and economic status. As previously reported, along with economic development, retinal diseases are the main reasons for blindness and low vision.^3^ Southern Jiangsu Province is one of the most developed and wealthy regions in China. Guan^4^ reported that the prevalence of blindness and low vision in adults aged ≥50 years were mainly caused by cataract, ocular fundus disease, refractive error, and cornea disease in Qidong City of Jiangsu. No blindness survey of elder people has been conducted in southern Jiangsu Province. The present study assessed the prevalence and causes of blindness of people aged >50 years in this region.

The study was performed to determine the primary reason for VI using a strictly epidemiological investigation method and baseline data for future eye care planning in southern Jiangsu Province were obtained.

## METHODS

***Census and samples: ***Residents in the southern Jiangsu Province were selected randomly using a stratified and clustered sampling technique with probabilities proportionate to the size of the population in each cluster. This survey sampled 10 towns and communities with a total of 46,000 households. The population comprised equal percentages (50%) of males and females.

Based on the eye disease survey program of nine provinces^5^, all tests were approved by the health department of Jiangsu Province and complied with the medical ethics standard. This study adhered to the Declaration of Helsinki. Ethics approval was obtained from the National Survey Ethics Committee. Written informed consents were obtained from all participants prior to enrollment, and verbal consent was obtained for the illiterate participants. After the examination, the patients were advised for further treatment. The selection method for the clusters was provided in the nine provinces that participated in the eye survey program.^6^ Using overall stratified random sampling, we randomly sampled 30 basic sampling clusters, 28 of which were formal investigation clusters, whereas the other two were considered as spare. The total population of the 28 survey clusters was 28,455, 6,722 or 23.6% of whom were aged ≥ 50 years. The sample size was calculated using the following parameters: estimated prevalence of blindness in people aged>50, 5.8%; worst acceptable estimate, 2.6%; design effect, 2.0; confidence level, 95%; expected response rate, 90%; and minimum population of people aged>50 years to provide the required sample size, 5,018.

Information on the visual and physical status was recorded by the village doctor. Clinical ophthalmology examinations were completed by nine ophthalmologists. All data were recorded in the unified format eye disease epidemiological survey forms.

***Ophthalmic Examination: ***To check the consistency test prior to the formal investigation, we selected a cluster of approximately 1,000 people for pre-experimentation. The pre-experiment determined whether the equipments were ready and whether investigators were prepared. A total of 249 samples were selected. The numbers of males and females were 249 and 318, respectively. The average ages of men and women were 62.0±9.1 years and 60.0±8.2 years, respectively. To determine the cause of VI, 100 individuals were selected for eye examination. Consistency test results showed that each Kappa value reached the investigation requirements.

Community activity centers, halls, and other places were used as the examination sites. An action inconvenient inspected object was adopted for home inspection by an ophthalmologist. Visual acuity (VA) was determined using a chart projector with tumbling E letters at a distance of 2.5 m. VA was recorded when the patient was able to read correctly at least three of the four letters in the smallest line. If the subject was unable to read the largest E letters in the chart (20/200 E letter) at 2.5 m, the participant was requested to walk nearer until he/she could read and recognize the letters. VA was calculated as VA=0.1×distance/2.5 (e.g., at 2 m, VA=0.1×2/2.5=0.08). Low vision was defined as the best-corrected VA <20/60 (6/18), but > 20/400 in the better eye.^7^ For data analysis, blindness or VI was defined in accordance with the best-corrected VA for the best eye. Blindness was defined as VA<3/60 in the better eye, with available spectacle correction, severe VI (SVI) as VA<6/60 to 3/60, and VI as VA<6/18 to 6/60.

Using clinical judgment, the ophthalmologist determined the principal cause of visual impairment or blindness for each eye. Faced with multiple factors, the ophthalmologist attempted to identify the most significant cause for the limitation of vision. Other contributory causes were specified by the ophthalmologist as secondary factors. In cases with different causes of visual reduction in a participant's both eyes, the diagnosis in the less affected eye was used.^8^ When two causes appeared to equally contribute to visual impairment, the primary cause was assigned to the one more responsive to treatment for the restoration of vision. Cataract was regarded as the main cause of severe low vision if the fundus was obscured by lens changes or if no evident fundus abnormalities were observed in eyes with significant cataract. Degenerative myopia was defined by a myopic refractive error of at least -6 diopters and typical myopic maculopathy with stretching of the macula.^9^ Retinal disease comprised diabetic retinopathy, retinal vascular disease, and age-related macular degeneration (AMD).^10^ Diabetic retinopathy was present if the macula showed cystoid macular edema, hard exudates, intraretinal hemorrhages, and microaneurysms.^10^ AMD was characterized by degeneration or dystrophy of macular retina without any other cause, such as diabetic or hypertensive retinopathy.^11^^,^^12^

***Statistical Analysis:*** Statistical analysis was performed using SPSS software version 17 (SPSS, Inc., Chicago, IL). χ^2^-test was performed. P values<0.05 were considered significant in all cases.

**Fig.1:**
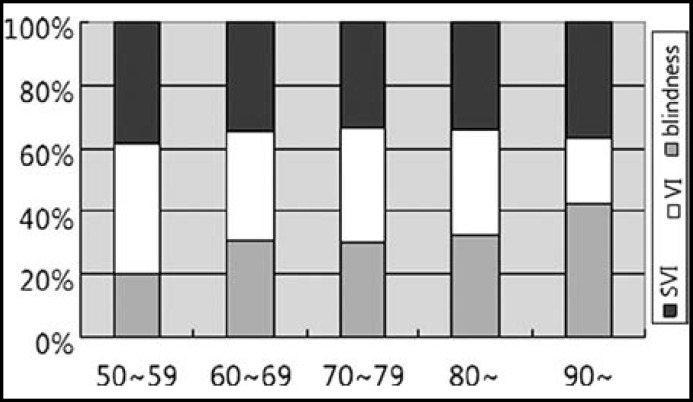
Prevalence of Blindness, Severe Visual Impairment (SVI) and Visual Impairment (VI) by age group

**Table-I:** The Prevalence of Blindness and Low Vision Age Composition

*Age*	*n*	*Unilateral blindness*		*Bilateral blindness*		*Unilateral low vision*		*Bilateral low vision*	
		*n*	*P(%)*	*n*	*P(%)*	*n*	*P(%)*	*n*	*P(%)*
50~	2187	18	0.82	5	0.23	40	1.83	7	0.32
60~	2601	83	3.19	9	0.35	84	3.23	21	0.81
70~	1035	59	5.70	16	1.55	57	5.51	33	3.19
80~	308	37	12.01	13	4.22	32	10.39	20	6.49
90~	19	4	21.05	4	21.05	1	5.26	3	15.79
Total	6150	201	3.27	47	0.76	214	3.48	84	1.37

*P=* *prevalence*

**Table-II:** Causes of Blindness, SVI and VI in Southern Jiangsu Providence

*Eye diseases*	*Blindness*		*SVI+VI*	
	*n*	*%*	*n*	*%*
Refractive error	*0*	*0*	*17*	*3.89*
amblyopia	*9*	*3.05*	*25*	*5.72*
cataract	*116*	*39.32*	*223*	*51.03*
Lens posterior capsule	*7*	*2.37*	*12*	*2.75*
Corneal opacity	*14*	*4.75*	*13*	*2.97*
atrophy eyeball	*28*	*9.49*	*1*	*0.23*
glaucoma	*5*	*1.69*	*5*	*1.14*
optic atrophy	*22*	*7.45*	*7*	*1.6*
AMD	*18*	*6.10*	*41*	*9.38*
high myopia macular degeneration	*31*	*10.51*	*41*	*9.38*
diabetic retinopathy	*2*	*0.68*	*9*	*2.06*
Retinal detachment	*3*	*1.02*	*2*	*0.46*
Retinal/ choroid disease	*11*	*3.73*	*11*	*2.52*
Other diseases	*24*	*8.14*	*13*	*2.97*
No reason	*5*	*1.69*	*17*	*3.89*

## RESULTS

This survey comprised 6,722 available participants. The number of participants evaluated in the actual survey was 6,150. The response rate was 91.5%. The numbers of males and females were 2,613 and 3,537, respectively. The average ages of men and women were 64.0±8.1 years and 63.1±9.3 years, respectively. The prevalence of blindness, SVI, and VI increased with age (Fig.1).

The prevalence rates of unilateral and bilateral blindness were 3.27% and 0.76%, respectively. The prevalence rates of unilateral and bilateral low vision were 3.48% and 1.37%, respectively (Table-I). In the group of 201 individuals with unilateral blindness, 55 elders had contralateral eye low vision.

A total of 295 eyes were blind. Cataract [(116 (39.32%)] was found to be the leading cause of blindness (Table-II). Atrophic eyeball [28 (9.49%)] was the main cause of blindness, closely followed by high myopia macular degeneration [31 (10.51%)]. A total of 437 eyes had SVI and VI. The top three causes of low vision were cataract, high myopia macular degeneration, and age-related macular degeneration. The numbers of cataract, high myopia macular degeneration, and age-related macular degeneration were 223 (51.0%), 41 (9.38%), and 41 (9.38%), respectively.

## DISCUSSION

According to reports of the WHO diagnostic criteria of blind and low vision, approximately 6.7 million people are blind and 7.1 million have low vision in China. China is emerging as one of the countries with very high incidence of blindness and low vision worldwide. In recent years, as a result of the developing demographic composition of the population and socio-economic status, the original prevalence data of eye diseases were not representative in China. With the use of the stratified cluster random sampling method, we conducted an eye disease epidemiological investigation in southern Jiangsu Province. Our survey determined the accurate reasons that caused blindness and low vision, especially the ocular fundus condition.

The prevalence rates of blindness, SVI, and VI in north Jiangsu Province were all remarkably lower than the estimated values by WHO in 2002^1^. These data showed that the prevalence rates of unilateral blindness, bilateral blindness, unilateral low vision, and bilateral low vision were 3.27%, 0.76%, 3.48%, and 1.37%, respectively. Additionally, individuals affected by blindness and low vision comprised 3.12% and 5.76%. Compared with a previous study^13^, the current study showed that the prevalence of unilateral blindness is at an average level. However, blind eyes had lower prevalence. The prevalence of bilateral blindness, binocular, and monocular low vision were markedly lower. The low prevalence of bilateral blindness may have been caused by the development of the local economy and well implementation of the policy for blindness prevention.^14^ However, higher rates of monocular blindness were found than previously reported. People could maintain normal life with another good eye, thus encouraging them to ignore the treatment of abnormal eyes. Analysis of the blindness and low vision in the different ages showed that the prevalence of these conditions gradually increased with increasing age. This result agreed with other investigations.^15^ Our survey reported that the number of adults aged >80 years with low vision was higher than those of other ages. Future efforts for the prevention of blindness will focus on older individuals.

The top three well known eye diseases that lead to blindness, which were also the primary causes of low vision, were cataract, high myopia macular degeneration, and eye atrophy. The results showed that future work concerning blindness prevention should be focused on prevention and cataract treatment. Contrary to the results from a previous study^16^, glaucoma and trachoma ceased to be the main diseases associated with blindness. High myopia macular degeneration and aged-macular degeneration have emerged as the primary causes of blindness. The result of this study was similar to the reports from Beijing^17^ and Shanghai.^18^ Aging population and economic progress were found to have led the increased prevalence of high myopia macular degeneration and aged-macular degeneration. At present, the economic development and percentage of aging population in the southern Jiangsu Province are all considered at a high level. Although the prevalence of AMD was lower than cataract, VI caused by AMD was unavoidable. Current options for prevention are limited. Additionally, the awareness on impairment is still insufficient. Considering the aging of the Chinese population, more attention should be focused on VI caused by AMD.

On one hand, we will continue to improve the coverage of cataract surgery anticipating a reduction of treatable blindness and low vision in the future. On the other hand, we need to improve eye disease diagnosis and treatment, especially for retinal diseases. We would also improve the diagnosis and treatment of eye diseases in the community hospital. Simultaneously, more attention must be paid to eye disease diagnosis and treatment training of general practitioners. The developed system should contain early detection and long-term development for the screening process as well as early treatment for high myopia macular degeneration, aged- macular degeneration, and diabetic retinopathy. Regular follow-up will effectively control and prevent vision damage.

The prevalence and main causes of blindness and low vision in southern Jiangsu Province have been elucidated. Results from this study has provided useful baseline information for future studies and intervention program. The need to target intervention programs for the prevention of visual impairment towards the older population is also emphasized.
